# Do ethnicity and gender influence posterior tibial slope?

**DOI:** 10.1007/s10195-017-0443-1

**Published:** 2017-02-02

**Authors:** Salvatore Bisicchia, Gavinca M. Scordo, Johan Prins, Cosimo Tudisco

**Affiliations:** 1 Ospedale San Pietro Fatebenefratelli, Rome, Italy; 2 Department of Orthopaedic Surgery, Sport Traumatology Unit, University of Rome “Tor Vergata”, Viale Oxford 81, 00133 Rome, Italy; 3 Life Little Company of Mary Hospital, Pretoria, South Africa

**Keywords:** Tibial slope, Ethnicity, Gender, Knee

## Abstract

**Background:**

Ethnicity and gender can affect posterior tibial slope; however, studies on this topic have limitations and are in disagreement. The aim of the present study was to evaluate posterior tibial slope in a large group of consecutive patients, determining whether ethnicity and gender can influence its value. Secondly, to determine intra- and inter-rater reliability of the two radiographic methods adopted.

**Materials and methods:**

Posterior tibial slope was calculated (rater 1) in lateral view X-rays of the knee according to the posterior tibial cortex (PTC) and tibial proximal anatomical axis (TPAA) methods. Data were matched with ethnicity and gender. For determination of intra- and inter-rater reliability, 50 random X-rays were selected, and blindly measured by two other raters (2 and 3).

**Results:**

A total of 581 radiographs were included (413 white and 168 black knees). Comparing white and black subjects, a statistically significant difference was found for both PTC (4.9 ± 1.2 vs 7.1 ± 2.9, *p* < 0.0001), and for TPAA (7.7 ± 1.1 vs 10.2 ± 3.0, *p* < 0.0001). In white subjects, an influence of gender was found only for TPAA (6.4 ± 1.1 in males vs 7.6 ± 1.1 in females, *p* < 0.0001). In black subjects, an influence of gender was found only for PTC (7.4 ± 3.0 in males vs 6.2 ± 2.9 in females, *p* = 0.01). Intra-rater reliability was good for both methods for rater 1, and very good for rater 2. Inter-rater reliability among the 3 raters was very good for both methods.

**Conclusions:**

Differences in posterior tibial slope between different ethnic groups exist. Differences observed between genders are conflicting and might be too small to have implications in clinical practice. The TPAA method is recommended for the evaluation of posterior tibial slope because of higher intra- and inter-rater reliability.

*Level of evidence 3* Case-control study.

## Introduction

Evaluation of the sagittal plane of the knee has recently gained popularity because its modification has effects on biomechanics and articular stability, greatly influencing the results of many knee procedures, such as anterior cruciate ligament reconstruction [1, 2], posterior cruciate ligament reconstruction, posterolateral corner reconstruction, high tibial osteotomy [3], unicondylar knee arthroplasty [4, 5], and total knee arthroplasty [6–12].

In the literature a wide range of values of posterior tibial slope has been reported because of a substantial inter-individual variability [11, 13, 14], and many different methods have been described. In all cases, a line tangent to the medial tibial plateau is traced, and the angle formed with the perpendicular direction to one of the following axes is considered: proximal tibial anatomical axis (PTAA) [15], anterior tibial cortex (ATC) [16], posterior tibial cortex (PTC) [17], proximal fibular anatomical axis (PFAA) [17], and fibular shaft axis (FSA) [17]. The same authors [17] compared all these techniques and stated that PTAA and PTC are the most reliable methods and are not influenced by age, sex, height, and weight. Cullù et al. [18] compared the various methods and found, for the same patients, higher values using PTC [16] and lower values with PFAA [17].

Ethnicity and gender are other variables that can affect the amount of posterior tibial slope [13, 19]. However, studies on this topic have important methodology limitations and their results are in disagreement. In fact, de Boer et al. [13] found a difference only between white and black subjects; on the other hand, Haddad et al. [19] found only an influence of gender.

Because the results from previous studies are conflicting, new research is needed to detect whether differences in posterior tibial slope exist between genders and ethnic groups. Therefore, the aim of the present study was to evaluate posterior tibial slope in lateral view X-rays in a large group of consecutive patients at two centers, determining whether ethnicity and gender can influence its value. Secondly, to determine intra- and inter-rater reliability of the two radiographic methods adopted.

## Materials and methods

The digital picture archival and communication systems (PACS) at two institutions were searched for all knee X-rays. All radiographs were anonymized to preserve patients’ privacy and labeled with their corresponding ID. Radiographs were excluded if femoral condyles were not perfectly superimposed on the lateral view, if they showed any tibial fractures (previous or actual), severe degenerative joint disease [defined as Kellgren-Lawrence ≥ 3 (29)], or a knee arthroplasty (total or unicondylar) or a high tibial osteotomy were previously done.

**Fig. 1:**
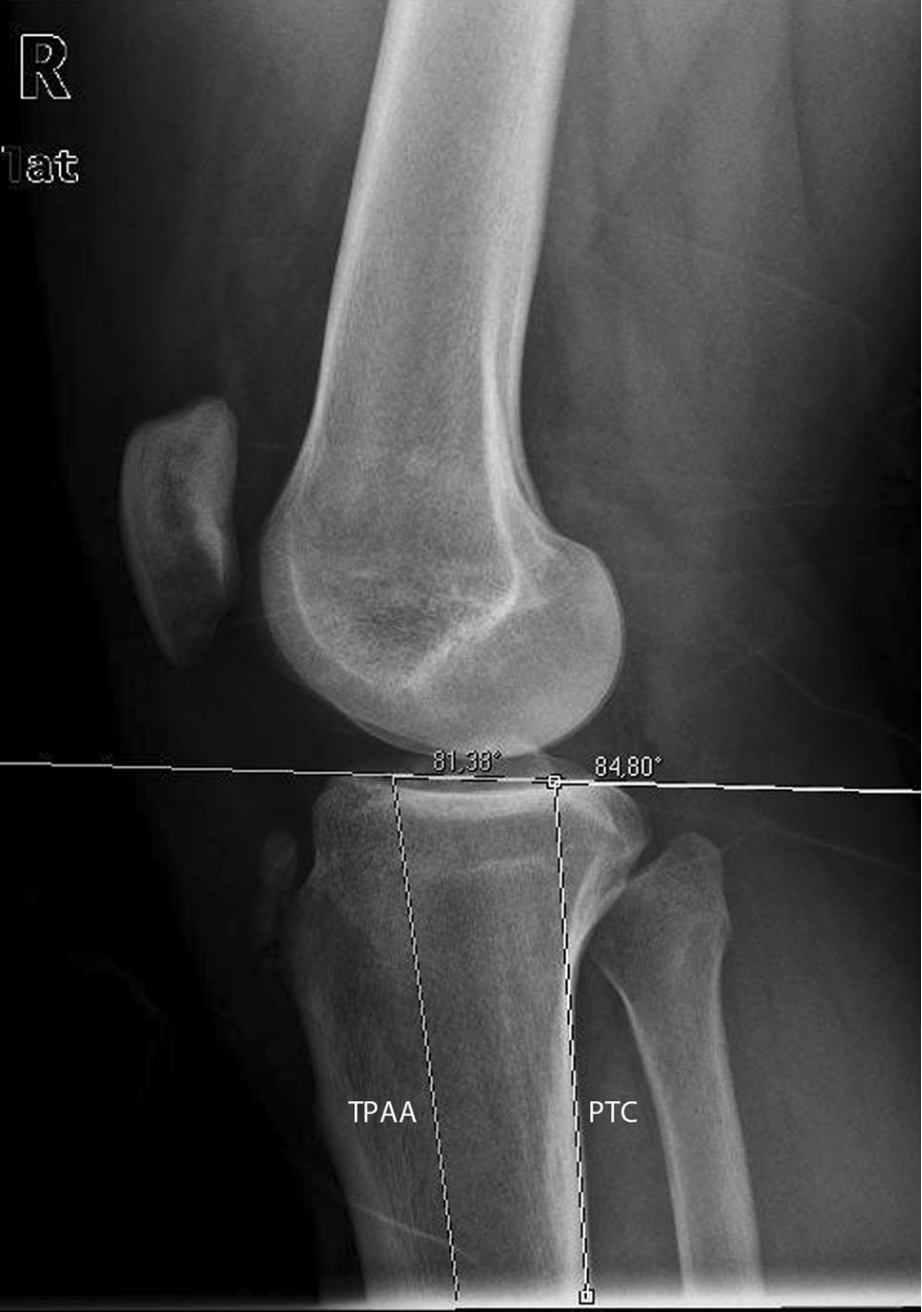
On a true lateral-view radiograph on the knee, the angle between the tangent to the medial tibial plateau and the perpendicular direction to the tibial proximal anatomical axis (TPAA) and posterior tibial cortex (PTC) were measured

All included images were uploaded on a digital viewer (Kodak Carestream, Carestream Health, Inc. Rochester, NY), and posterior tibial slope was evaluated using PTC [17] and TPAA [15]. These methods were chosen because they are the most reliable methods in determining posterior tibial slope, and are not influenced by age, sex, height, and weight of the patients [17]. On every lateral view knee radiograph, the angle between the tangent to the medial tibial plateau and the perpendicular direction to PTC [17] and TPAA [15] were measured (Fig. 1). In case X-rays of both knees of the same patient were available in the databases, only one was considered for analysis (randomly either the right or the left). Furthermore, degenerative changes were reported according to Kellgren-Lawrence [20]. All the data were recorded on an Excel spreadsheet (Microsoft Office, Redmond, WA). The authors performing radiographic measurements were blinded to clinical and demographic data of the patients.

Once all radiographic data were collected, clinical files of the patients were reviewed and age, height, weight, gender, and ethnicity were extracted. Also in this case, personal data were anonymized and clinical files were numbered with their corresponding ID. Patients were divided into different sub-groups: white, black, and Asian. PTC and TPAA values in each group were compared to determine whether ethnicity had an influence on their value, and between male and female patients in the same sub-group to determine whether gender had an influence on their value.

### Statistical methods

For determination of intra-rater (test–retest) reliability and inter-rater reliability in the measurement of posterior tibial slope, a statistician was consulted to determine how many patients and raters would be necessary to reach significance. For determination of sample size, the formula of the correlation coefficient of Bravais ($r = \frac{{\sigma_{xy} }}{{\sigma_{x} \sigma_{y} }}$) was used. After some algebraic passages, it is possible to obtain the formula $n = \left( {\frac{{Z_{1 - \alpha } + Z_{\beta } \sqrt {1 - r^{2} } }}{r}} \right)^{2} + 2$. In previous studies [19, 21, 22] a good agreement (with a correlation coefficient *r* ≥ 0.7) was reported. Supposing that in the present study only moderate agreement (*r* = 0.5) would be obtained, considering an error *α* = 5%, and a power 1−*β* = 90%, it was determined that 40 patients should be evaluated by each observer to reach significance. For these reasons, it was deemed that 50 randomly extracted patients would be more than enough for determination of intra- and inter-rater reliability. On the other hand, it is not possible to calculate “a priori” the number of raters needed; but in previous studies, 2–3 raters were used [19, 21, 22]. For these reasons, 3 raters were involved: a general orthopedist (rater 1), a fellowship-trained sports medicine orthopedic surgeon (rater 2), and an Associate Professor of Orthopedics and Traumatology with more than 25 years of experience in knee surgery and sports medicine (rater 3). Intra-rater reliability was evaluated for raters 1 and 2, who evaluated twice the same 50 random radiographs 4 months apart (test–retest reliability). For inter-rater reliability, rater 3 evaluated once the same 50 radiographs.

Descriptive statistics were used to summarize the characteristics of the study group and sub-groups. An unpaired *t*-test was used to compare continuous variables, while a chi-square test was used for categorical variables. Two-sided statistical significance was defined as *p* < 0.05. Post-hoc power analysis was also performed. To determine whether statistically significant differences were also clinically relevant, size effect was determined using Cohen’s d coefficient: values >0.2 were considered small, >0.5 medium, >0.8 large, and >1.3 very large [23]. The intraclass correlation coefficient (ICC) was used to assess intra- and inter-observer reliability. An ICC < 0.20 was considered poor agreement, 0.20–0.40 fair agreement, 0.41–0.60 moderate agreement, 0.61–0.80 good agreement and 0.81–1.00 very good agreement [24]. Statistical analyses were performed with SPSS v.15.0 (SPSS Inc., an IBM Company, Chicago, IL, USA).

## Results

From a pool of more than 3000 consecutive X-rays, a total of 581 radiographs were included in this study. There were 413 white (71%) and 168 black (29%) knees, no Asian patients were available for this study. Demographic data are listed in Table 1. In the whole group of 581 patients, the mean PTC and TPAA were 6.0 ± 1.6 and 8.6 ± 2.2 degrees, respectively, with no statistically significant differences in terms of gender distribution, age, height, weight, and degenerative changes between white and black groups (Table 1).

**Table 1:** Demographic data in white and black subjects

	White	Black	*p*
Gender distribution (M:F)	228 M (55%):185 F (45%)	91 M (54%):77 F (46%)	>0.05
Age (years)	43.2 ± 17.7	40.3 ± 15.2	>0.05
Height (cm)	172.0 ± 11.3	170.1 ± 13.4	>0.05
Weight (Kg)	69.2 ± 6.8	68.1 ± 6.6	>0.05
OA grading	1.7 ± 1.1	1.9 ± 1.3	>0.05

Comparing white and black subjects, a statistically significant difference was found for both PTC and TPAA (Table 2). Sub-group analysis in patients of the same gender confirmed a statistically significant difference in posterior tibial slope in patients from different ethnicities, with higher PTC and TPAA angles for black subjects in both genders (Table 3). Further sub-group analysis provided conflicting results on the influence of gender in patients of the same ethnicity. In fact, in white subjects, an influence was found only for TPAA, while in black subjects it was found only for PTC (Table 4).

**Table 2:** Comparison of posterior tibial slope determined with posterior tibial cortex (PTC) and tibial proximal anatomical axis (TPAA) methods in white and black patients

	White	Black	*p*	Power	Cohen’s d
PTC	4.9 ± 1.2	7.1 ± 2.9	<0.0001	100%	0.99
TPAA	7.7 ± 1.1	10.2 ± 3.0	<0.0001	100%	1.37

**Table 3:** Sub-group analysis showing the influence of ethnicity on the amount of posterior tibial slope with posterior tibial cortex (PTC) and tibial proximal anatomical axis (TPAA) methods in patients of the same gender

	White	Black	*p*	Power	Cohen’s d
Male					
PTC	5.0 ± 1.2	7.4 ± 3.0	<0.0001	100%	1.05
TPAA	6.4 ± 1.1	10.5 ± 3.1	<0.0001	100%	1.76
Female					
PTC	5.1 ± 1.2	6.2 ± 2.9	<0.0001	99.7%	0.50
TPAA	7.6 ± 1.1	9.9 ± 3.0	<0.0001	100%	1.02

**Table 4:** Sub-group analysis showing the influence of gender on the amount of posterior tibial slope with posterior tibial cortex (PTC) and tibial proximal anatomical axis (TPAA) methods in patients of the same ethnicity

	Male	Female	*p*	Power	Cohen’s d
White					
PTC	5.0 ± 1.2	5.1 ± 1.2	>0.05	–	–
TPAA	6.4 ± 1.1	7.6 ± 1.1	<0.0001	100%	1.1
Black					
PTC	7.4 ± 3.0	6.2 ± 2.9	0.01	75%	0.41
TPAA	10.5 ± 3.1	9.9 ± 3.0	>0.05	–	–

Intra-rater (test–retest) reliability was good for both methods for rater 1, and very good for rater 2. Inter-rater reliability among the 3 raters was very good for both methods. The TPAA method achieved higher ICC for both intra- and inter-rater reliability (Table 5).

**Table 5:** Intra-rater (test–retest) reliability and inter-rater reliability (measured by intraclass correlation coefficient — ICC) for posterior tibial cortex (PTC) and tibial proximal anatomical axis (TPAA) methods obtained by repeated measurements in 50 random radiographs

	PTC	TPAA
Intra-rater (rater 1)	0.67	0.79
Intra-rater (rater 2)	0.89	0.93
Inter-rater (among 3 raters)	0.81	0.88

## Discussion

The main finding of the present study was the detection of differences in posterior tibial slope between white and black subjects.

Ethnicity and gender have already been reported to affect the value of posterior tibial slope [13, 19, 25, 26]. However, there are important limitations in these studies. The methodology on bare tibias from cadavers [13, 25] is not feasible in the clinical setting; furthermore, in one study, about 40% of mixed subjects were included in the gender analysis, that were excluded from the ethnicity analysis [13]. On the other hand, Haddad et al. [19] included only MRIs done for ligament and cartilage problems, obtaining a sample with features that are potentially different from the general population; they also included a small mixed group (5%) in gender analysis, which was excluded from ethnic analysis. Moreover, the results of these studies are in disagreement: de Boer et al. [13] found a difference only between white and black subjects; on the other hand, Haddad et al. [19] found only an influence of gender, that other studies were not able to detect [13, 26].

In the current study, ethnicity was considered and statistically significant differences were found for PTC and TPAA between white and black subjects (Table 2). Sub-group analysis in patients of the same gender confirmed a statistically significant difference in patients from different ethnicities, with higher angles for black subjects in both genders (Table 3). Further sub-group analysis provided conflicting results on the influence of gender in patients of the same ethnicity. In fact, in white subjects, an influence was found only for TPAA, while in black subjects it was found only for PTC (Table 4).

In previous studies, many authors reported a wide range of values in posterior tibial slope because of a substantial inter-individual variability [4, 7, 10, 11, 14]. A wide range of posterior tibial slope was also found in the present study, but with a very small standard deviation, indicating that the majority of patients have a posterior tibial slope that approximates the averages of this study, and only a limited number of subjects require particular attention in the evaluation and pre-operative planning (in particular black subjects).

In our study, intra-rater (test–retest) reliability was good for both methods for rater 1, and very good for rater 2. Inter-rater reliability among the 3 raters was very good for both methods. The TPAA method achieved higher ICC for both intra- and inter-rater reliability (Table 5), and this method was therefore recommended. ICCs for intra- and inter-rater reliability in this study are in agreement with previously published results [19, 21, 27, 28].

This study has some strengths, such as a large sample size, gender and ethnicity were considered as independent variables, the methods adopted for determination of posterior tibial slope were chosen because they are the most reliable and are not influenced by sex, age, height, and weight; intra- and inter-rater reliability were determined in a random group of 50 radiographs that were reviewed blindly by 3 raters.

Limitations exist for this study. Posterior tibial slope was measured on conventional 2D radiographs instead of using a 3D imaging technique, such as CT/MRI. Previous studies have reported that there is a good correlation between X-rays and CT/MRI, with an average error of about 3.4° [22, 27]. Because plain X-rays are cheap and easy to obtain, they are routinely adopted as a first line diagnostic modality in patients undergoing knee imaging for any reasons worldwide. Moreover, plain X-rays are the only imaging modality ordered in clinical practice in high tibial osteotomy or knee arthroplasty. Furthermore, it was deemed that there would be a smaller proportion of pathological X-rays in the databases, compared to MRI that are performed as a second line imaging modality to confirm knee disorders. For these reasons, it was supposed that a true lateral X-ray of the knee (with femoral condyles perfectly superimposed) would compensate for rotations of the tibia and would provide all the information needed for the determination of posterior tibial slope in most clinical scenarios, being cheap, ready to use, and having a worldwide spread. Another limitation of this study is the absence of Asian and/or Hispanic patients. Asian patients have been reported to have a posterior tibial slope that is significantly steeper compared to white and black subjects [19, 25]. Previous studies [11, 14] found no correlations between posterior tibial slope and degenerative joint disease. In the current study, severe degenerative joint disease was an exclusion criterion, and a subgroup analysis between knees with and without osteoarthritis was not done.

More studies are needed in the future to better evaluate the influence of gender, ethnicity, or other variables on posterior tibial slope.

In conclusion, differences in posterior tibial slope between different ethnic groups exist. Differences observed between genders are conflicting and might be too small to have implications in clinical practice. The TPAA method is recommended for the evaluation of posterior tibial slope for its higher intra- and inter-observer reliability.
